# Improving MR sequence of 18F-FDG PET/MR for diagnosing and staging gastric Cancer: a comparison study to ^18^F-FDG PET/CT

**DOI:** 10.1186/s40644-020-00317-y

**Published:** 2020-06-16

**Authors:** Dong Zheng, Yi Liu, Jiajin Liu, Ke Li, Mu Lin, Holger Schmidt, Baixuan Xu, Jiahe Tian

**Affiliations:** 1 Department of Nuclear Medicine, Chinese People’s Liberation Army General Hospital, 28 Fuxing Road, Haidian Street, Beijing, 100853 China; 2 Department of Radiology, Chinese People’s Liberation Army Strategic Support Force Characteristic Medical Center, Beijing, 100101 China; 3 Department of General Surgery, The Seventh Medical Center of Chinese People’s Liberation Army, General Hospital, Beijing, 100010 China; 4 MR Collaboration, Diagnostic Imaging, Siemens Healthineers Ltd, Shanghai, 201318 China; 5 MR Education, Customer Services, Siemens Healthcare GmbH, 91052 Erlangen, Germany

**Keywords:** FDG, PET/MR, PET/CT, HASTE, BLADE and gastric cancer

## Abstract

**Purpose:**

Evaluate the feasibility of fluorine-18 (^18^F) fluorodeoxyglucose (FDG) positron emission tomography (PET) and magnetic resonance (MR) imaging in patients with gastric cancer by optimizing the scan protocol and to compare the image quality to ^18^F FDG PET and computed tomography (CT).

**Methods:**

The PET/CT and PET/MR imaging were sequentially performed in 30 patients with gastric cancer diagnosed by gastroscope using a single-injection-with-dual-imaging protocol. After intravenous injection of ^18^F-FDG (mean, 249 MBq), PET/CT imaging including low-dose CT was performed (mean uptake time, 47 ± 6 min), and PET/MR imaging including a T1-weighted Dixon sequence for attenuation correction and two different T2-weighted sequences was subsequently acquired (88 ± 15 min after ^18^F-FDG injection). Four series of images (CT from PET/CT, T1W, T2W Half-Fourier acquisition single-shot turbo spin-echo [T2W-HASTE] and T2W-BLADE from PET/MR) were visually evaluated using a 3–4 points scale for: (1) image artifacts, (2) lesion conspicuity and (3) image fusion quality. The characteristics of the primary lesions were assessed and compared between the PET/CT and PET/MR acquisitions.

**Results:**

The image quality and lesion conspicuity of the T2W-HASTE images were significantly improved compared to that of the T2W-BLADE images. A significantly higher number of artifacts were seen in the T2W-HASTE images compared with the T1W and CT images (*p* < 0. 05). No differences in the accuracy of image fusion between PET/MR and PET/CT (*p* > 0. 05); however, significant difference was seen in the lesion conspicuity measurements (*p* < 0.05) with T2W-HASTE being superior. For information about the primary lesion characteristics, the T2W-HASTE images provided the most successful identifications compared with those of the T1W and PET/CT (13*vs*7*vs*5) images.

**Conclusions:**

PET/MR with the T2W-HASTE was better at revealing the details of local stomach lesions compared with PET/CT imaging. Combining the PET/MR with the T2W-HASTE technique is a promising imaging method for diagnosing and staging gastric cancer.

## Introduction

Gastric cancer is one of the most important malignant tumors affecting the health and quality of human life worldwide and especially in Asia [1, 2]. It has become a prime focus of clinical and imaging research. Various imaging technologies play critical roles in gastric cancer screenings, treatment strategy designs, assessment of prognosis and curative effect.

Traditional imaging methods have been in use for a long history, including abdominal and gastroscopic ultrasound, spiral computed tomography (CT), magnetic resonance imaging (MRI), and positron emission tomography–computed tomography (PET/CT). PET/CT can provide quantitative measurement of metabolic activity at the molecular level and yield local anatomical details as well [3] and thus has received clinicians’ attention quickly [4]. However, PET/CT also has its disadvantages, such as low soft tissue sensitivity/resolution and increasing the patient’s exposure to ionizing radiation (6.40–19.70 mSv) during the examination [5] – particularly significant for more vulnerable population namely children and women at reproductive age [6] - and poor detectability of soft tissue in CT scans.

In 2011, the United States and the European Union approved simultaneous PET/MR scanners for clinical application [7]. PET/MRI has proven to be a superior diagnostic imaging method. Combining the metabolic parameters of PET scanning with the excellent soft tissue imaging quality of MRI, the weakness of PET/CT in clinical diagnosis has been alleviated. MRI can also provide additional functional information, such as diffusion-weighted imaging or spectral analysis. A systematic review by Riola-Parada et al. [8] showed that PET/MRI was superior or similar to PET/CT for the diagnosis of more than 16 different neoplastic lesions. However, at present, the literature still supports PET/CT as a main player in the diagnosis and staging of gastric cancer [9–11], and PET/MRI has rarely been reported as being used in the study of gastric tumors, which is partially because of the confounding effects from respiratory movements, gastrointestinal peristalsis and cardiac pulsation on MRI. Nevertheless, driven by the appealing merits of high soft tissue contract and no ionizing radiation, MRI is widely used in abdominal imaging. Many important developments in MR technology have been achieved in recent years, like higher field strengths, more powerful gradient imaging system, newer and faster three-dimensional gradient echo technologies, parallel imaging technologies, and multi-channel phase array coils, which makes possible high-quality clarity and high spatial resolution MR imaging within a single breath-holding time range [12].

Using MRI, the T2-weighted (T2W) protocols have been the most important sequences used in the diagnosis and staging of gastric cancer. However, due to the lengthy acquisition time, T2W sequences are exceptionally sensitive to motion. Previous studies have concluded that applying the T2W-BLADE sequence enhanced image contrast, improved image quality and reduced the sensitivity to motion artifacts [13, 14], In addition, the T2W echo-planar fast spin echo HASTE can also reduce motion artifacts in T2W images. It employs a wide receiver bandwidth, a narrower space of the Radio Frequency (RF) refocusing pulses resulted in negligible artifacts with sub second temporal resolution. Based on the above advantages, T2W-HASTE sequence can be used to examing of acute abdomen disorders [15], and cholelithiasis [16], and in the elderly and children [17]. However, the HASTE sequence results in echo signal attenuation that leads to reduction in image signal-to-noise ratio (SNR).

In this study, we first optimized the image quality of T2W-HASTE and T2W-BLADE for patients with gastric cancer by balancing the sequence parameters including TR/TE, matrix size and turbo factor and we compared the diagnostic potential of these two protocols. T2W-HASTE and T2W-BLADE were then compared in regards of image quality and lesion conspicuity, and the one with better performance was included in the PET/MR protocol. Finally, the feasibility of PET/MRI in patients with gastric cancer was evaluated and compared to PET/CT in terms of lesion characteristics.

## Materials and methods

### Patient population

Our prospective study was approved by the institutional review board. Patients included in this study were: (a) those newly diagnosed with gastric cancer on endoscopic biopsies, (b) those who consumed food and water normally and tolerated stomach filling, (c) those who gave their written informed consent for study enrollment, and (d) those who agreed to have PET/MRI and PET/CT examinations performed at the Nuclear Medicine Department of our hospital. Exclusion criteria were: (a) history of malignant tumors, abdominal surgeries, or abdominal inflammation, (b) those with contraindications for MRI and PET scans, such as those with cardiac pacemakers or severe diabetes.

All patients included in our study underwent both whole-body [18F]-fluorodeoxyglucose (^18^F-FDG)-PET/CT and upper-abdominal ^18^F-FDG-PET/MRI on the same day in our nuclear department between December 2016 and November 2017.

### ^18^F-FDG pet/CT

All patients fasted for at least 6 h and then rested quietly for 20–30 min before intravenous administration of ^18^F-FDG (produced in our institute) at a dose of 4.4 MBq (0.12 mCi)/kg of body weight. Hyoscine butylbromide (10 mg, Chengdu No. 1 Drug Research Institute Company Limited, Chengdu, China) was injected intramuscularly 10 min before PET/CT examination. Then all patients were asked to drink 800 ~ 1000 mL of physiological saline immediately prior to PET/CT acquisition to distend the stomach. PET-CT imaging was performed with a hybrid PET-CT scanner (Biograph Truepoint 64; Siemens Medical Solutions, Knoxville, TN, USA). CT images (5 mm slices) were obtained from the chin to the upper thigh at 120 kVp and 100 ~ 120 mA. Emission PET images were obtained over the same anatomic range after the administration of ^18^F-FDG (mean interval, 47 ± 6 min), with 15–25 min/5 to 7 beds depending on patient weight and height and using the three-dimensional acquisition mode. CT images were reconstructed with 3 mm thickness.

### ^18^F-FDG pet/MR

PET/MR was acquired after completion of PET/CT using residual ^18^F-FDG from the initial injection (mean interval, 20.0 ± 5.7 min). All patients were also asked to drink 600 ~ 800 ml of water immediately prior to the examination. Patients were trained to hold their breath and breathe normally before PET/MR scans. PET/MRI examinations were performed using an integrated PET/MRI scanner (Biograph mMR; Siemens Healthcare, Erlangen, Germany) which had PET detector rings placed inside a 3 T MRI gantry with a standard six-channel-phased-array body matrix coil. The field of view was set from the diaphragmatic dome to the level of renal hilum. In our protocol, both high resolution T2W-HASTE and T2W-BLADE were acquired without fat saturation. T2W-HASTE was acquired under free-breathing; T2W-BLADE sequence was acquired with respiratory trigger. The scan parameters of each sequence were summarized in Table 1. We also performed diffusion-weighted imaging (DWI) using a single-shot echo-planar imaging sequence with b-values of 0, 200, 800 mm^2^/s. The scan time for dedicated stomach MR imaging is approximately 15 min while PET images were acquired simultaneously for 5 min in one bed position.

**Table 1:** MRI Parameters

	T2W-HASTE	T2W-BLADE	DWI	T1-VIBE-dixon
TR (ms)	2680	3000	8600	4.04
TE (ms)	83	119	72	1.24/2.47
Slice thickness (mm)	3	2	5	3
Slice gap (mm)	0.6	0.4	1	0.6
FOV (mm)	236 × 320	380 × 380	270 × 360	328 × 420
Matrix	378 × 512	320 × 320	82 × 110	188 × 320
Flip angle(°)	140	80	–	12
Echo chain length	378	38	82	–
Bandwidth	610	505	1976	1040
Fat suppression	–	–	+	–
NEX	1	–	3	1
Voxel size (mm^3^)	0.6 × 0.6 × 3.0	1.2 × 1.2 × 2.0	3.3 × 3.3 × 5.0	1.8 × 1.3 × 3.3
BLADE coverage	–	100%	–	–

*HASTE* Half-Fourier acquisition single-shot turbo spin-echo, *VIBE* volumetric interpolated breath-hold examination, *TR* repetition time, *TE* echo time, *FOV* field of view, *NEX* number of excitations

### Evaluation of FDG-PET/CT and PET/MR

The acquired CT, MR and PET images were sent to a dedicated reading workstation (Syngo.via; Siemens Healthcare, Erlangen, Germany), which allows for simultaneous review of the MRI, CT, PET as well as fused PET/MRI and PET/CT images.

First, two physicians, including one experienced radiologist (17 years in MR, 21 years in CT, and 5 years in nuclear medicine) and one experienced nuclear medicine physician (15 years in nuclear medicine and 19 years in diagnostic imaging, including 16 years in MR and 19 years in CT), evaluated all images together to determine the location of primary gastric cancer, either as a consensus or with reference back to the gastroscopy results. The criteria for the positive primary gastric cancer lesions were as follows: (1) localized thickening of gastric wall on the structural images, and/or (2) abnormal increases in ^18^F-FDG uptake beyond the adjacent gastric wall on PET images [18] (diffuse physiologic uptake by the normal gastric wall thickness was excluded).

Second, the T2W-HASTE and the T2W-BLADE images were independently evaluated regarding image artifacts and lesion conspicuity by the two physicians. A 3-point scale was used for assessment of image artifacts [19] (1 - artifacts of hampering image evaluation, 2 - little artifacts of not hampering image evaluation, and 3 - no artifacts). The lesion conspicuity was evaluated using a 4-point scale [20] - 1 (less than 25% of lesion borders definable), 2 (25–50% of borders definable), 3 (50–75% of borders definable), 4 (more than 75% of lesion borders definable).

Third, T1W and CT images were independently examined by the two physicians and compared to T2W images (select the high-scoring sequence in HASTE and BLADE through the previous step), the image artifacts and lesion conspicuity were evaluated according to the above criteria. Additionally, image fusion quality was evaluated using a 3-point scale according to degrees of matching between the anatomic structure of the lesion and the metabolic increased region (for HASTE or BLADE & DIXON, the PET from PET/MR was used; for CT, the PET from PET/CT was used) [21], namely, 1 - matching poor, 2 - matching moderate, and 3 - matching excellent.

Lastly, another assessment was done for coexistent findings beneficial to the diagnosis and staging of positive lesions, including lesion shape, stratification, and invasion of adjacent tissue structures. The PET/CT dataset was first reviewed by the two readers in consensus. To avoid memory bias, after an interval 2 weeks, the PET/MR dataset was then reviewer by the same readers.

### Statistical analysis

All statistical analyses were performed using SPSS Statistics Version 21 (IBM, Armonk, NY, USA) software. Weighted kappa (k) statistics were used to assess interobserver agreement between the two physicians’ evaluations for each imaging modality, a kappa value of < 0.20, between 0.21–0.40, 0.41–0.60, 0.61–0.80, and 0.81–1.00 were considered poor, fair, moderate, good, and excellent, respectively. Wilcoxon matched-pairs signed sum ranks test was used to compare the image artifacts and lesion conspicuity scores between the T2W-HASTE and T2W-BLADE images. The Kruskal-Wallis H test was used to compare the image artifact grades, image fusion qualities, and lesion conspicuities in the PET/MR-T2W, T1W, and PET/CT images. A *P*-value < 0.05 was considered statistically significant.

## Results

Our study recruited 30 patients (age range, 34–76 years; mean, 58 ± 10.5 years). There were 24 men (age range, 34–76 years; mean, 58 ± 10.4 years) and 6 women (age range, 44–70 years; mean, 55 ± 11.3 years). PET/MR and PET/CT were used to evaluate 30 cases of gastric cancer. Among these, one case had no positive primary lesion on PET and structural images, so the primary lesion was detected by gastroscopy to be located in the gastric antrum. One case was PET negative, but had a local gastric wall thickening and central depression on the T2 image and CT images; later it was clinically found to be a crater-like primary lesion. The primary lesion locations in the 30 patients were as follows: nine were found in the gastric fundus, ten in the antrum, 11 in the gastric body. Twenty-five out of 30 patients had an ulcerative tumor type and the remaining had a bulge or mass tumor type. For the PET part, PET/CT and PET/MR revealed the same number of lesions. For PET/CT, the average SUVmax was 7.79, ranging from 1.5 to 38.1; For PET/MR, the average SUVmax was 6.99, ranging from 1.05 to 34.3. Measured in T2W-HASTE images, the average maximum length diameter was 4.57 cm, ranging from 0.5 cm to 11.2 cm. The ratings of the two physicians’ results are shown in Table 2.

**Table 2:** The scores of image artifacts, lesion conspicuity, fusion quality in three modalities(n)

Modality	image artifacts			fusion quality			lesion conspicuity			
	1	2	3	1	2	3	1	2	3	4
PET/CT										
Reader1	0	1	29	0	8	22	6	19	5	0
Reader2	0	2	28	0	9	21	10	16	4	0
PET/MR-T1WI										
Reader1	0	13	17	2	9	19	6	15	9	0
Reader2	0	14	16	1	9	20	4	15	10	1
PET/MR-T2WI HASTE										
Reader1	0	26	4	1	17	12	1	12	15	2
Reader2	0	26	4	1	19	10	1	12	13	4
PET/MR-T2WI BLADE										
Reader1	11	18	1	–	–	–	8	12	10	0
Reader2	10	19	1	–	–	–	8	14	8	0

### The interobserver agreement

The interobserver agreement was excellent for the TW2-BLADE images regarding image artifacts and lesion conspicuities (kappa [k] = 0.912,k = 0.865, respectively) and excellent for the T2W-HASTE images regarding image artifacts, lesion conspicuity, and image fusion quality (k = 1, k = 0.891, k = 0.868. respectively). The interobserver agreement was also excellent for the T1W images regarding image artifacts and image fusion quality (k = 0.933, k = 0.931, respectively), and excellent for the PET/CT images regarding image fusion quality (k = 0.918). The interobserver agreement was moderate for the T1W images regarding lesion conspicuity (k = 0.785) and the PET/CT images regarding image artifacts and lesion conspicuity (k = 0.651, k = 0.767, respectively) as shown in Table 3.

**Table 3:** The inter observer agreement between two readers (Kappa value)

	PETCTTT	PETMR-T1W T1WI	PETMR-T2W-HASTEEEEEEE	PETMR-T2W-BLADEDEDEE
image artifacts	0.651	0.933	1	0.912
conspicuity conspicuity	0.767	0.785	0.891	0.865
fusion quality	0.918	0.931	0.868	–

### Comparison of the image artifacts and lesion conspicuity between T2W-HASTE and T2W-BLADE sequences

**Fig. 1:**
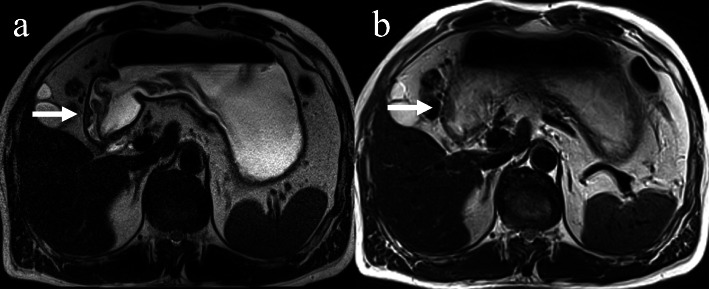
Comparison of two T2-weighted MR images of a patient with ulcerative moderately to poorly differentiated adenocarcinoma at greater curvature of the gastric antrum showing superiority of HASTE (**a**) versus BLADE (**b**). Localized thickening of gastric antrum wall can be seen (arrow). While some artifacts are present in both images, image artifacts were rated as “not hampering image evaluation” (score 2) for T2- HASTE, but rated as“hampering image evaluation” (score 1) for T2-BLADE. Lesion conspicuity was rated as “excellently delimitable” (score 4), and“less than 25% of lesion borders definable” (score 1), respectively. In addition, T2- HASTE image showed an ulcer with crater shape, and the submucosa high signal line, which is of great value for diagnosis and staging, but not on T2-BLADE image

**Fig. 2:**
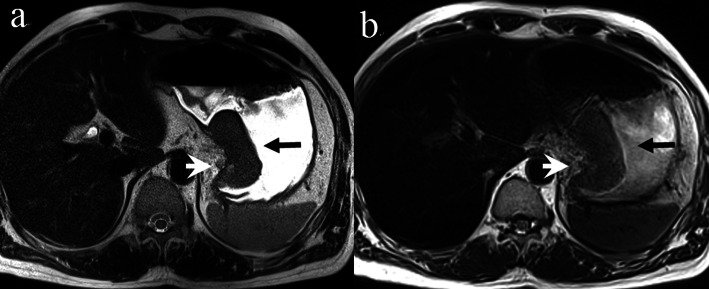
Comparison of two T2-weighted MR images of a patient with protrude moderately differentiated adenocarcinoma involving the lesser curvature of the stomach showing the superiority of HASTE (**a**) over BLADE (**b**). The intermediate-signal-intensity gastric mass can be seen on both images (arrow). Image artifacts were rated as“not hampering image evaluation” (score 2) for T2- HASTE, but rated as“hampering image evaluation” (score 1) for T2-BLADE. Lesion conspicuity was rated as“excellently delimitable” (score4), and “50–75% of borders definable” (score3), respectively. HASTE image showed the outer boundary remained smooth and a small, round lymph node next to the lesion (arrowhead), while due to the artifacts of BLADE image, there appeared to be an irregular/nodular outer border with perigastric fat infiltration (arrowhead), which may lead to incorrect staging

There were many cases of artifacts for both sequences, especially the BLADE. In about 10 cases the observation of the primary lesion was affected in the BLADE images. In the HASTE images, the observation of primary lesion was not affected, and there was significant difference in the score of artifacts between the two sequences(*P* < 0.01). The HASTE images showed only one 1-point case regarding lesion conspicuity caused by a negative primary focus, and had three full-score cases, while the BLADE images showed eight 1-point cases and no full-score cases. The difference in lesion conspicuity score between the two sequences was statistically significant (*P* < 0.05) (show in Table 4). From the above results, we concluded that the image quality of HASTE was improved compared to the BLADE. Therefore, the HASTE protocol was selected for the subsequent PET/MR-T2W image studies (Figs. 1 and 2).

**Table 4:** Comparison of image artifacts and lesion conspicuity between two T2W sequences (score, median, upper and lower quartile)

MR sequence	image artifacts	lesion conspicuity
T2WI-HASTE	2 (2, 2)	3 (2, 3)
T2WI-BLADE	2 (1, 2)	2 (1, 3)
Z	−3.249	−2.502
*P*	0.00	0.012

**Fig. 3:**
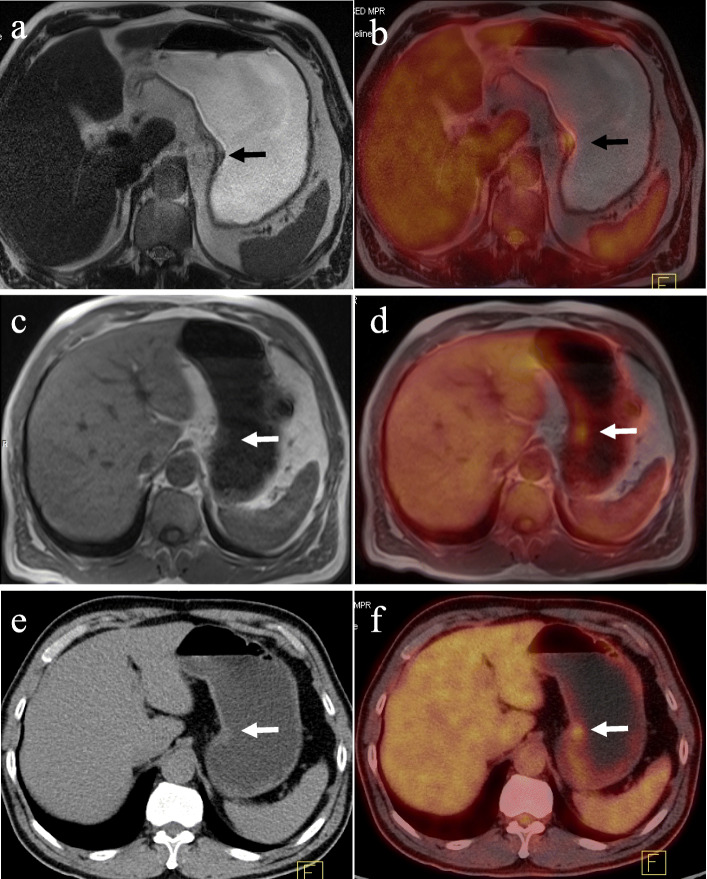
A 58-year-old man with gastric carcinoma at the lesser curvature of the stomach (arrow). Image artifacts were rated as“not hampering image evaluation” (score 2) and lesion conspicuity was rated as“excellently delimitable” (score4) for T2-HASTE image (**a**). T2-HASTE image showed an ulcer with crater shape and intact high-signal-stripe layer, which is a characteristic information of great value for staging, while this was not clearly visible on MRI-T1W and PET/CT images. Image artifacts were rated as “no artifacts” (score 3), lesion conspicuity was rated as“25–50% of borders definable” (score2) for both MRI-T1W (**c**) and CT images (**e**), image fusion quality was rated as“excellent” (score3) for PET/MR-T2W (**b**), T1W (**d**) and PET/CT (**f**) images

Comparison in terms of image artifacts, lesion conspicuity and image fusion quality of PET/MR-T2W-HASTE (since it revealed a higher score than T2W-BLADE in the previous analysis), PET/MR-T1W and PET/CT images (Fig. 3)

#### Comparison of image artifacts

The artifacts among the three modal images were significantly different (*P* < 0.01) as shown in Tables 2 and 5. Most of the PET/CT images (Physician 1, 29; Physician 2, 28), and more than half of the T1W images (Physician 1, 17; Physician 2, 16) had no artifacts. In contrast, most of the PET/MR-T2W images (Physician 1, 26; Physician 2, 26) had artifacts, and only four cases had no artifacts; three cases were located in the gastric antrum and one case in the stomach body.

**Table 5:** Comparison of image artifacts, lesion conspicuity and image fusion quality in three modalities (score, median, upper and lower quartile)

Modality	image artifacts	image fusion	lesion conspicuity
PET/CT	3 (3, 3)	3 (2, 3)	2 (2, 2)
PET/MRT1	3 (2, 3)	3 (2, 3)	2 (2, 3)
PET/MRT2	2 (2, 2)	2 (2, 3)	3 (2, 3)
Х^2^	39.01	9.275	14.696
*P*	**0.00**	0.055	**0.023**

#### Comparison of image fusion quality

Most of the PET/CT images showed an excellent match without a poor match. Most of the PET/MR-T1W images showed excellent matches well, but with three examples of poor matching degree. Most of the PET/MR-T2W images showed excellent or medium matching degree with two examples of poor matching degree. No statistical significance was found when comparing the accuracy of fusion among the three modal images(P>0.05) (shown in Tables 2 and 5).

#### Comparison of lesion conspicuity

There was a statistically significant difference in the conspicuity between the three modal images(*P* < 0.05) as shown in Tables 2 and 5. The conspicuity scores of PET/CT images were the lowest with mostly 2-point (few 1-point, no 4-point). The conspicuity scores of T1W images were mainly 2-point – (few 3-point, one 4-point). PET/MR-T2W images got the highest scores with 3-point, (many 2-point, six 4-point).

### Assessment for coexistent findings

**Fig. 4:**
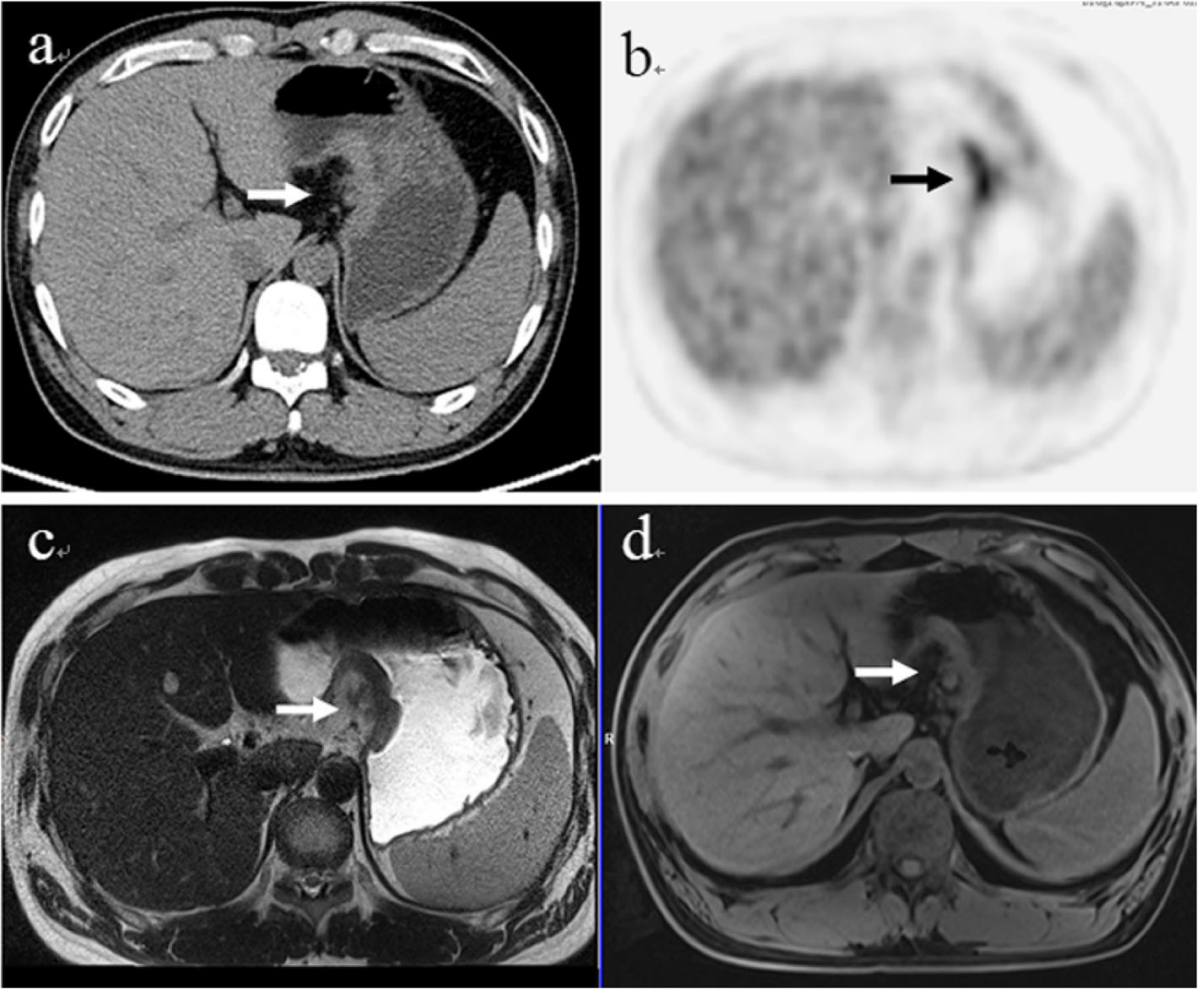
A 34-year-old man with gastric carcinoma at the lesser curvature of the stomach. A nodular exudation of the gastric wall (arrow) was visible in PET/CT (**a**, **b**) as well as in T2-HASTE (**c**) and MRI-T1W (**d**) images, which is the characteristic information of great value for staging

Of the 29 positive primary lesions, characteristic information including stratification, nodular exudation of the gastric wall and crater shape of the lesion was mostly revealed using the T2W-HASTE images (stratification 5, nodular exudation of the gastric wall 4, crater shape of the lesion 4), followed by T1W-VIBE-DIXON (nodular exudation of the gastric wall 4, crater shape of the lesion 1), and among these, only 5 cases were found on PET/CT images (nodular exudation of the gastric wall 4, crater shape of the lesion 1) (Figs. 3 and 4). Therefore, the PET/MR images were superior to the PET/CT images in being able to show lesion details (13 vs. 5) .

## Discussion

PET/MRI has been widely touted as a promising diagnostic imaging technique. MRI has intense soft tissue contrast, high signal versatility, high functional/physiologic capabilities, and low radiation exposure [22]. Presently, frequent reports have shown that PET/MRI is useful for diagnosing and staging cancers. Even though PET/CT imaging yields better pulmonary nodule detection [23], PET/MRI performs as well as PET/CT in the diagnosis of head and neck tumors [24], prostate cancer [25], and multiple myeloma [26], and PET/MRI is better than PET/CT imaging in the diagnosis of breast cancer [27], colorectal cancer [28], liver cancer [29], abdominal occasional tumors [30], gynecologic tumors [31], lymphoma [32], and bone [33] and brain metastases [34].

Despite that high-speed MR techniques could overcome some of the limitations of MR in detecting gastric cancer, such as motion artifacts, MR has not yet been widely accepted as a standard imaging method for gastric cancer staging. Therefore, there is no consensus of gastric MRI scanning scheme [35, 36]. T1-weighted gradient echo sequences with fat suppression, FSE or TSE T2-weighted images, steady-state precession true fast imaging (True-FISP), diffusion-weighted imaging (DWI), T1-weighted imaging with three-phase dynamic enhancement all play a role in detecting gastric cancer. A review article [37] published this year has shown that the accuracy of MRI is similar or slightly better when compared to the currently most frequently used imaging modalities (i.e. EUS and CT) in the evaluation of T-staging. However, its limited availably and higher costs would only make MRI an alternative imaging modality when CT is contraindicated or when CT results are ambiguous. Thus this study considers the facts that dynamic enhanced scanning is quite tedious and can cause discomfort or enhance risk of allergy in patients, and that the longer time of DWI scanning does not solve the inherent problem of the influence of motion artifact on the images. Additionally, a study has shown a non-enhanced MR scan can display 3–7 slices of normal gastric wall and T2-weighted MR imaging may make it possible to noninvasively assess the depth of mural invasion by gastric carcinomas [38]. Therefore, we proposed to combine non-enhanced MR scans with PET in the study so as to speed up the acquisition time, reduce the cost, improve the availability. As mentioned earlier, T2W-HASTE and T2W-BLADE are currently the most commonly used T2W sequences for gastric MR examinations. HASTE uses a continuous 150 to 180 degree refocusing phase pulse after a 90-degree pulse excitation to complete all of the signal acquisition and a half-Fourier-leaf technology acquisition. This significantly shortens the acquisition time, which in turn leads to reduced motion artifacts. It employs a wide receiver bandwidth resulted in negligible chemical shift artifacts, and it also adopted a narrower space of the RF refocusing pulses with sub second temporal resolution to effectively minimize susceptibility artifacts. However, the HASTE sequence also has its drawbacks. Because it only has one excitation pulse, all images are obtained by a long echo chain, increasing the change of T2 relaxation between rows in k-space. This results more pixel graininess due to obvious echo signal reduction that leading to decreased SNR. In this study, we retained the fast acquisition of HASTE for the gastric images and optimized parameters, including a prolonged TR, increased matrix, increased turbo factor, which resulted in significantly improved spatial resolution with a pixel value up to 0.6 × 0.6 × 3.0 mm while maintaining the scan time below 1 s per slice.

BLADE sequence acquires data of k-space with rotating parallel lines instead of parallel lines which can dramatically reduce the occurrence of motion and magnetic susceptibility artifacts [39]. However, the disadvantage of the blade artifact correction technique is the increased scan time, which partially reduces the advantages of the sequence in motion correction.

In this study, we compared the image artifacts and the lesion conspicuity of the two sequences. The results showed that the HASTE sequence was superior to the BLADE sequence in these two aspects. It indicated that high acquisition speed was the greatest advantage in gastric MR imaging. Although BLADE uses multiple strategies to decrease image artifacts, such as the Periodically Rotated Overlapping Parallel Lines with Enhanced Reconstruction (PROPELLER) technique to rotate and overlap data acquisition, diaphragmatic navigation, and respiratory gating, it was still beat by HASTE sequence in restraining artifacts. To be noted, our in-house optimization of HASTE sequence parameters significantly contributed to compensate for the low SNR. Especially in PET/MR imaging, PET and MR images are synchronously collected, and PET data must be collected in the free breathing state. Correspondingly, if the MR sequence (like the HASTE) is also collected under free breathing, it might improve the fusion of PET and MR images. Therefore, HASTE is a promising sequence to play an important role in the diagnosis and staging of gastric cancer using PET/MR.

This study revealed that PET/CT, PET/T1W and PET/T2W-HASTE showed similar results in the degree of image fusion but significantly different results in image artifacts: T2W-HASTE had the largest number of artifacts of the three image types. T2W-HASTE has relatively longer acquisition time (about 50 s) compared to T1W-VIBE-DIXON (about 20 s) and CT (about 5 s), so it is more vulnerable to diaphragmatic motion, stomach peristalsis and cardiovascular pulsation. Despite these results artifacts, T2W-HASTE still shows the highest score in terms of lesion conspicuity because of its high spatial resolution and good tissue contrast. As a result, the PET/MR protocol including a T2W-HASTE sequence can more comprehensively provide the characteristic information of the lesion than PET/CT (PET/MR vs PET/CT = 13 vs 5). This characteristic information included gastric wall stratification, nodular exudation of gastric wall and crater shape of the lesion, which was critical in the determination of location and staging of gastric cancer.

Limitations of the present pilot study were the small number of patients and the different timelines used in the PET imaging sessions with PET/MRI being performed on average 20 min following the PET/CT imaging. We will address these limitations in future studies with a larger number of participants and randomized scanning order between the PET/MRI and PET/CT imaging.

## Conclusion

By optimizing the scanning parameters of T2W-HASTE sequence, we achieved high spatial resolution while maintaining high scanning speed and high soft tissue resolution. Moreover, performing the scans in a breath-free state has advantages for the fusion of the PET images. Although the T2W-HASTE images had more artifacts in the quality comparisons of the PET/CT and PET/MRI images, T2W-HASTE images do not affect the degree of image fusion and provide more details regarding local stomach lesions. PET/MRI with the T2W-HASTE technique holds promise in diagnosing and staging gastric cancer.

## Data Availability

The datasets used and/or analyzed during the current study are available from the corresponding author on reasonable request.

## Abbreviations

FDG: Fluorodeoxyglucose
PET: Positron-emission tomography
MR: Magnetic resonance
CT: Computerized tomography
HASTE: Half-Fourier acquisition single-shot turbo spin-echo
RF: Radio Frequency
SNR: Signal-to-noise ratio
TR: Relaxation time
TE: Echo time
NEX: Number of excitations
FOV: Field of view
VIBE: Volumetric interpolated breath-hold examination
DWI: Diffusion-weighted imaging
